# Nucleic acid detection and molecular traceability of equine herpesvirus 1 in imported horses

**DOI:** 10.3389/fmicb.2026.1900100

**Published:** 2026-08-03

**Authors:** Lin Wang, Zhiqiang Gao, Zilong Bai, Jing Pu, Xiju Shi, Wei Zhang, Zihan Xu, Zhengliang Li

**Affiliations:** Science and Technology Research Center of China Customs, Beijing, China

**Keywords:** detection, equine herpesvirus type 1, imported horses, molecular traceability, nucleic acid detection

## Abstract

**Introduction:**

Equid alphaherpesvirus 1 is a major pathogen threatening the global equine industry, causing respiratory disease, abortion, and neurological disease.

**Methods:**

In April 2024, during routine pathogen screening of a batch of imported quarantine horses from the Netherlands, four out of 20 nasal swab samples tested positive for EHV-1 nucleic acid.

**Results:**

The affected horses exhibited only mild respiratory signs. ORF30 SNP typing at nucleotide position 2254 and Sanger sequencing confirmed that all positive samples belonged to the N752 non-neuropathogenic genotype. As virus isolation was unsuccessful, the ORF30, ORF33, and ORF34 gene fragments were amplified and sequenced directly from the positive samples. The complete identity of these gene sequences across all four samples indicated that they were caused by the same strain. Bayesian phylogenetic analysis based on concatenated multi-sequence alignment revealed that all reference strains first divided into two distinct clades according to host range: Clade A encompassed *Equus caballus*, *Equus ferus*, and *Equus asinus*, whereas Clade B was strictly restricted to *Equus caballus*. The strain identified in this study (EHV-1/China/2024) was located within the broadly host-adapted Clade A and formed a monophyletic group with an *Equus ferus*-derived strain isolated in Ireland in 2017 (MZ318706) with high posterior probability, which subsequently clustered with a sister group comprising another Irish *Equus ferus*-derived strain and a UK *Equus caballus*-derived strain, collectively constituting a predominantly European-derived evolutionary cluster.

**Discussion:**

While the horses were imported from the Netherlands, the evolutionary background of the virus pointed to the Ireland/British Isles region, suggesting that the strain may have entered the Netherlands via the European equine trade network before being introduced. This study reveals the molecular characteristics and phylogenetic position of EHV-1 carried by imported horses and uncovers a geographic discordance between the evolutionary background of the virus and its direct country of origin, providing molecular evidence for understanding the international transmission dynamics of EHV-1 and pathogen surveillance in cross-border equine movements.

## Introduction

Equid alphaherpesvirus 1 (EHV-1), commonly known as equine herpesvirus type 1, is the most economically and clinically significant equine herpesvirus (Hu et al., 2022; Słońska and Cymerys, 2025). EHV-1 belongs to the genus *Varicellovirus* within the subfamily *Alphaherpesvirinae* and is distributed worldwide in equine populations (Matczuk et al., 2018; Mesquita et al., 2021). It causes a variety of clinical syndromes, including mild to severe respiratory disease, abortion, neonatal foal death, chorioretinitis, and neurological disease commonly referred to as equine herpesvirus myeloencephalopathy (EHM) (Pusterla et al., 2022). Primary infection is established through the respiratory epithelium, mainly via close contact with infectious animals and inhalation of virus, and occasionally through contaminated feed and water (Kydd, 2021; Soboll-Hussey et al., 2024). Infection is followed by cell-associated viremia, which disseminates the virus to secondary target organs. The pathogenesis of EHM involves an inflammatory cascade triggered by infection of the vascular endothelium of arteries in the central nervous system (CNS), ultimately leading to local hemorrhage, thrombosis, and ischemia (Negussie et al., 2017). Between 2006 and 2007, a marked increase in severe EHM outbreaks in North America and Europe prompted the United States Department of Agriculture to classify it as a potentially emerging disease (Lunn et al., 2009). Given its significant impact, EHV-1 infection is listed as a notifiable disease by the World Organization for Animal Health (WOAH) to enable appropriate measures to ensure the safety of international trade in horses (Gifford et al., 2021).

The EHV-1 genome is a linear double-stranded DNA molecule of approximately 150 kbp. The genome consists of a unique long (UL) region and a unique short (US) region, flanked and separated by inverted repeat elements (TRL/IRL and TRS/IRS) (Kubacki et al., 2021; Tau et al., 2024). Conventionally, the genome has been annotated to contain 80 open reading frames (ORFs), encoding 76 distinct proteins and four duplicated proteins (Huang et al., 2002; Schnabel et al., 2019). Among these genes, ORF30, which encodes the catalytic subunit of the viral DNA polymerase, is closely associated with neuropathogenicity (Yamada et al., 2008). A single nucleotide polymorphism (A→G) at nucleotide position 2254 of ORF30 results in an amino acid substitution from asparagine (N) to aspartic acid (D) at residue 752 of the DNA polymerase, giving rise to two genotypes: N752 (non-neuropathogenic) and D752 (neuropathogenic) (Nishimura et al., 2024; Sutton et al., 2020). Numerous studies have demonstrated a strong correlation between the D752 genotype and EHM outbreaks, and SNP typing at this locus has therefore been widely used for preliminary assessment of the pathogenic risk of EHV-1 strains (Bryant et al., 2018; Gryspeerdt et al., 2010). However, global molecular epidemiological data on EHV-1 remain insufficient, particularly with regard to the molecular characterization and precise source tracing of strains carried by imported horses in the context of international horse movements.

In April 2024, during routine pathogen screening of a batch of imported horses undergoing quarantine, EHV-1 nucleic acid was detected in samples from four horses exhibiting only mild respiratory signs. Preliminary ORF30 SNP typing confirmed that all positive samples belonged to the N752 non-neuropathogenic genotype. To clarify the genetic characteristics of this strain and trace its potential origin, the ORF30, partial ORF33, and ORF34 gene fragments from the positive samples were amplified, sequenced, and subjected to phylogenetic analysis. This study aimed to reveal the phylogenetic position of this strain and its genetic relationship with circulating strains from both domestic and international sources, so as to provide a scientific basis for the molecular surveillance of cross-border EHV-1 transmission.

## Materials and methods

### Experimental materials

The samples used in this study were 20 nasal swabs collected in April 2024 from imported quarantine horses originating from the Netherlands, and were retained by our laboratory for future use. The sampling tubes were vortexed thoroughly and left to stand at room temperature for 1 min, after which 200 μL of the sample suspension was aspirated. Nucleic acids were extracted using the Viral Genomic DNA/RNA Extraction Kit (DP315) from Tiangen Biotech (Beijing) Co., Ltd., strictly following the manufacturer’s instructions. The nucleic acids were finally eluted in 60 μL of nuclease-free water and stored at 4°C for subsequent PCR amplification. All procedures involving clinical samples were conducted in a biosafety level 2 (BSL-2) laboratory.

### Detection and amplification primers

Specific primers and probes targeting the ORF33 (gB) gene of EHV-1 were designed for viral nucleic acid detection. Allele-specific real-time PCR primers and probes were also designed for SNP typing at nucleotide position 2254 of the ORF30 gene. Additionally, specific primers were designed to amplify fragments of the ORF33, ORF30, and ORF34 genes of EHV-1. The primer and probe sequences, amplicon sizes, and annealing temperatures are detailed in Table 1.

**TABLE 1:** Primers and probes used for real-time PCR and PCR.

Target	Primer name and sequence (5′–3′)	Amplicon length	Annealing temperature (°C)
ORF33 (*gB*)	EHV1gB-F: GGGGTTCTTAATTGCATTCAGACC	88 bp	55
	EHV1gB-R: GTAGGTGCGGTTAGATCTCACAAG		
	EHV1gB-P: [FAM]-TCTCCAACGAACTCGCCAGGCTGTACC-[BHQ1]		
2254SNP	EHV1-FW: TCTGGCCGGGCTTCAAC	56 bp	58
	EHV1-RE: TTTGGTCACCCACCTCGAA E1PG2254: [FAM]-ATCCGTCGACTACTCG-[BHQ1]		
	E1PA2254: [HEX]-ATCCGTCAACTACTCG-[BHQ2]		
ORF33 partial gene	FC2: CTTGTGAGATCTAACCGCAC	1181 bp	60
	RC: GGGTATAGAGCTTTCATGGG		
ORF30 partial gene	F8:GTGGACGGTACCCCGGAC	380 bp	60
	R2: GTGGGGATTCGCGCCCTCACC		
ORF34 partial gene	34F:GGCCCCAAGGATATTTAAGC	855 bp	58
	34R:GTTTGAGGCGGTTACGTCAG		

### Real-time quantitative PCR detection

The PCR reaction mixture was prepared using TaKaRa HS Taq DNA polymerase and dNTP mixture, with a total volume of 25 μL. The reaction mixture contained: 1× PCR Buffer, 0.2 mmol/L dNTP, forward primer EHV1gB-F and reverse primer EHV1gB-R at a final concentration of 0.3 μmol/L each, FAM-labeled EHV1gB-P probe at a final concentration of 0.1 μmol/L, 1.25 U of HS Taq DNA polymerase, and 5 μL of nucleic acid template. Samples with a Ct value ≤ 35 and a typical S-shaped amplification curve were considered positive, while those without a specific amplification curve were considered negative. Amplification and detection were performed on a Roche LightCycler 480 real-time quantitative PCR instrument using the following program: 95 °C for 3 min; followed by 45 cycles of 95 °C for 15 s, 55 °C for 10 s, and 60 °C for 30 s, with fluorescence signal collection in the FAM channel during the 60 °C step of each cycle.

### SNP site multiplex real-time PCR typing

The reaction mixture had a total volume of 25 μL and was prepared using TaKaRa HS Taq polymerase and accompanying reagents. The reaction mixture contained: 1× PCR Buffer, 0.2 mmol/L dNTP, forward primer EHV1-FW and reverse primer EHV1-RE at a final concentration of 0.4 μmol/L each, FAM-labeled E1PG2254 probe (detecting the D752 genotype, i.e., G2254) and HEX-labeled E1PA2254 probe (detecting the N752 genotype, i.e., A2254) at a final concentration of 0.2 μmol/L each, 1.5 U of HS Taq DNA polymerase, and 5 μL of nucleic acid template. Amplification was performed on a Roche LightCycler 480 real-time quantitative PCR instrument using the following program: 95 °C for 3 min; followed by 45 cycles of 95 °C for 15 s and 58 °C for 1 min, with simultaneous fluorescence signal collection in the FAM and HEX channels during the 58 °C step of each cycle. Amplification products positive by real-time PCR were subjected to Sanger sequencing using the Thermo Fisher Scientific BigDye Terminator v3.1 sequencing kit to verify the nucleotide type at the SNP site and ensure the accuracy of the typing results.

### Gene fragment amplification and sequencing

Target gene fragments were amplified in a 50 μL reaction system prepared with TaKaRa HS Taq DNA polymerase and dNTP reagents. The reaction mixture contained: 1× PCR Buffer, 0.2 mmol/L dNTP, each amplification primer at a final concentration of 0.5 μmol/L, 2.5 U of HS Taq DNA polymerase, and 10 μL of nucleic acid template. Amplification was performed on an Applied Biosystems 9700 PCR instrument using the following program: 94 °C for 5 min; followed by 45 cycles of 94 °C for 1 min, annealing at the corresponding temperature for 1 min, and 72 °C for 90 s; with a final extension at 72 °C for 7 min. Amplification products were identified by 1.5% agarose gel electrophoresis or using a QIAGEN QIAxcel capillary electrophoresis system, with products showing a single clear band of the expected size considered positive. Positive amplification products were purified by gel extraction and subjected to Sanger sequencing using the Thermo Fisher Scientific BigDye Terminator v3.1 sequencing kit on a Beijing Yuewei Gene GenReader 7010 genetic analyzer.

### Sequence processing and phylogenetic analysis

The ORF30, ORF33, and ORF34 sequences obtained in this study were manually curated and assembled. In addition to the EHV-1 strain sequence obtained in this study, 46 EHV-1 reference sequences published globally were retrieved from GenBank (GenBank accession numbers, strain names, countries of isolation, years of isolation, and host information are detailed in Table 2). The ORF30, ORF33, and ORF34 gene sequences corresponding to each strain were extracted. Multiple sequence alignment of all sequences was performed using MUSCLE in codon mode, and the three aligned gene fragments were concatenated using AMAS.

**TABLE 2:** Information of EHV-1 reference sequences used for phylogenetic analysis.

No.	Accession	Country	Host	Collection_Year
1	KR021354	USA	*Equus caballus*	2010
2	KT324724	Australia	*Equus caballus*	1977
3	KT324725	Australia	*Equus caballus*	2007
4	KT324727	Australia	*Equus caballus*	2003
5	KT324729	Australia	*Equus caballus*	2002
6	KT324730	Australia	*Equus caballus*	1994
7	KT324731	Australia	*Equus caballus*	1993
8	KT324732	Australia	*Equus caballus*	1990
9	KT324733	Australia	*Equus caballus*	1982
10	KT324734	Australia	*Equus caballus*	1977
11	KU206465	United Kingdom	*Equus caballus*	1982
12	KU206467	Hong Kong SAR, China	*Equus caballus*	1984
13	KU206440	United Kingdom	*Equus asinus*	2003
14	KU206425	United Kingdom	*Equus caballus*	2013
15	KY852346	United Kingdom	*Equus caballus*	2016
16	MN912433	India	*Equus caballus*	2014
17	MW396756	India	*Equus caballus*	2007
18	MW396757	India	*Equus caballus*	2013
19	MW396758	India	*Equus caballus*	2014
20	MW396759	India	*Equus caballus*	2017
21	MW396760	India	*Equus caballus*	2018
22	MW855958	Belgium	*Equus caballus*	2021
23	MW855961	France	*Equus caballus*	2021
24	MZ318700	Ireland	*Equus ferus*	2009
25	MZ318701	Ireland	*Equus ferus*	2002
26	MZ318702	Ireland	*Equus ferus*	2006
27	MZ318704	Ireland	*Equus ferus*	2010
28	MZ318705	Ireland	*Equus ferus*	2013
29	MZ318706	Ireland	*Equus ferus*	2017
30	MZ357402	Switzerland	*Equus caballus*	2021
31	OP271695	Kazakhstan	*Equus caballus*	2011
32	OR085496	USA	*Equus caballus*	2005
33	OR801338	China	*Equus caballus*	2021
34	PP084325	Argentina	*Equus caballus*	1999
35	PP084326	Argentina	*Equus caballus*	2007
36	PP481413	Italy	*Equus caballus*	2023
37	PP839875	Morocco	*Equus caballus*	2010
38	PP856396	Belgium	*Equus caballus*	1997
39	PP856397	Belgium	*Equus caballus*	2003
40	PQ014267	China	*Equus caballus*	2023
41	PQ630625	USA	*Equus caballus*	2017
42	PQ630626	USA	*Equus caballus*	2021
43	PQ630627	USA	*Equus caballus*	2023
44	PQ630628	USA	*Equus caballus*	2024
45	PV960828	Kazakhstan	*Equus caballus*	2015
46	PZ019512	South Korea	*Equus caballus*	2012

The XML configuration file for Bayesian evolutionary analysis was constructed using BEAUti v1.10.4 (Drummond et al., 2012), with the best-fit substitution model selected, an uncorrelated relaxed clock with lognormal distribution, and the Bayesian Skyline coalescent model. Bayesian analysis was performed using BEAST v1.10.4 (Baele et al., 2025), with the MCMC chain run for 1 × 10^8 generations, sampling every 10,000 generations. Tracer v1.7.1 was used to examine the effective sample size (ESS) values of all parameters, ensuring that all ESS values exceeded 200, indicating adequate convergence of the MCMC chain. The first 10% of trees were discarded as burn-in, and TreeAnnotator v1.10.4 was used to generate a maximum clade credibility (MCC) tree, summarizing the posterior estimates of node heights and 95% highest posterior density (HPD) intervals. Finally, the phylogenetic tree was visualized and refined using FigTree v1.4.4 for genetic evolution and source tracing analysis of the strains.

## Results

### EHV-1 nucleic acid detection

**FIGURE 1:**
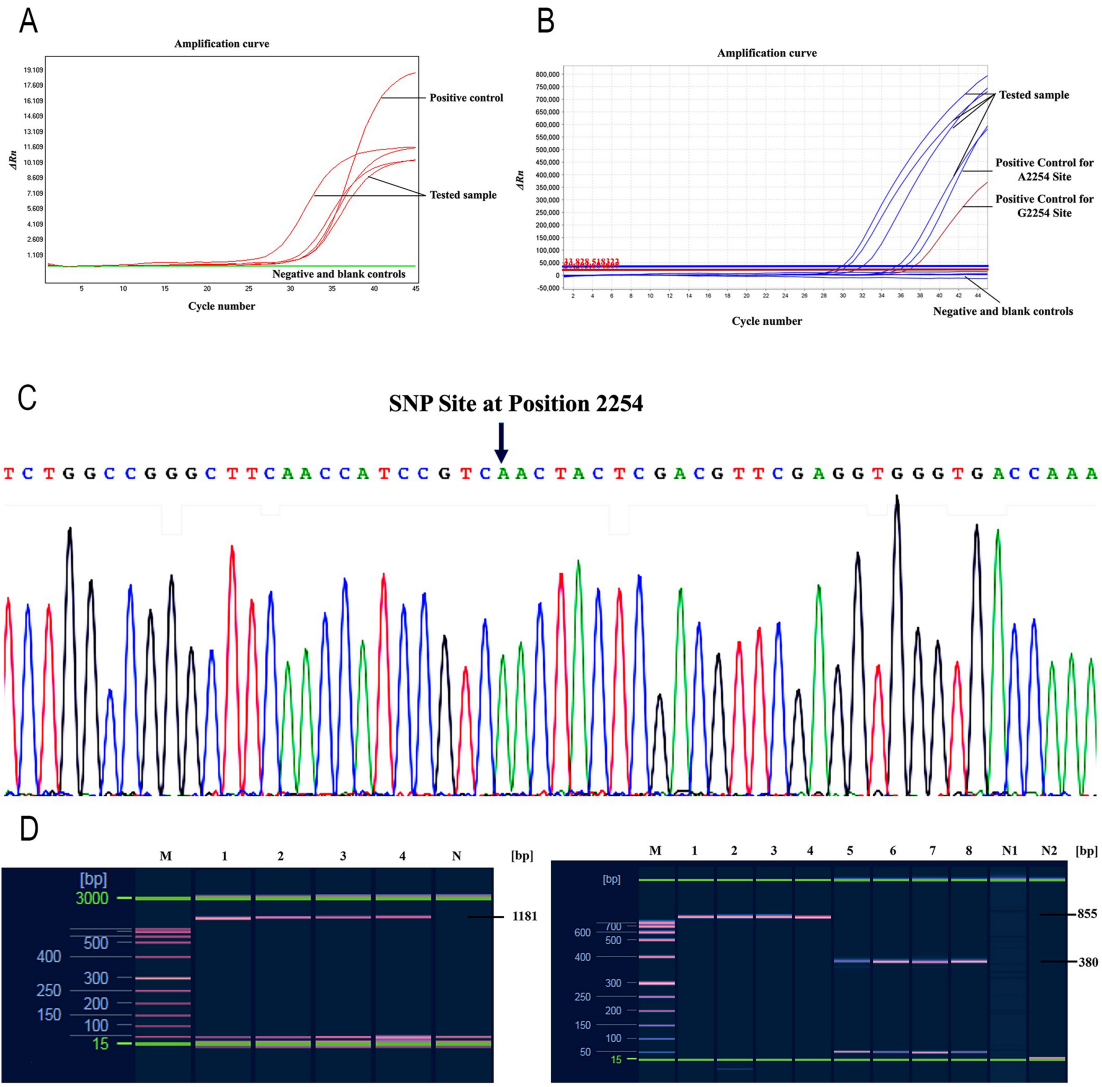
Detection and molecular characterization of EHV-1 in nasal swab samples from imported horses. **(A)** Real-time quantitative PCR amplification curves of the ORF33 gene. Positive samples (*n* = 4) displayed typical S-shaped amplification curves, while negative samples (*n* = 16) showed no specific fluorescence signal. The dashed line indicates the threshold. **(B)** Allele-specific multiplex real-time PCR typing of the ORF30 nucleotide position 2254. All four positive samples exhibited fluorescence signals exclusively in the HEX channel (A genotype, N752), with no signal detected in the FAM channel (G genotype, D752). NTC, no-template control. **(C)** Sanger sequencing chromatograms confirming the A genotype at ORF30 position 2254 in all four positive samples. The arrow indicates the SNP site. **(D)** Gel electrophoresis of PCR products amplified with specific primers targeting ORF33, ORF30, and ORF34.

In April 2024, a total of 20 nasal swab samples were collected from a batch of imported horses undergoing quarantine. Real-time quantitative PCR targeting the ORF33 gene was used for EHV-1 screening, with a Ct value ≤ 35 considered positive. The results showed that 4 out of the 20 samples were positive for EHV-1 nucleic acid, all displaying typical smooth amplification curves with marked increases in fluorescence signal. The remaining 16 samples showed no specific amplification curves and were considered negative (Figure 1A).

For the four nucleic acid-positive samples, allele-specific multiplex real-time PCR typing at nucleotide position 2254 of the ORF30 gene was further performed. The results revealed that all positive samples carried the A genotype at this site (Figure 1B). This was further confirmed by Sanger sequencing, verifying that all EHV-1-positive strains detected in this study belonged to the N752 genotype and were classified as non-neuropathogenic strains (Figure 1C).

The ORF33, ORF30, and ORF34 genes of the four positive samples were amplified using specific primers, all yielding specific bands of the expected sizes (Figure 1D). Following Sanger sequencing, sequence curation, and multiple sequence alignment, BLAST homology analysis demonstrated that the ORF33, ORF30, and ORF34 gene sequences of the four positive samples were completely identical, indicating that these samples were caused by the same EHV-1 strain. The consensus sequence was used for subsequent phylogenetic and source tracing analyses.

### Phylogenetic analysis

**FIGURE 2:**
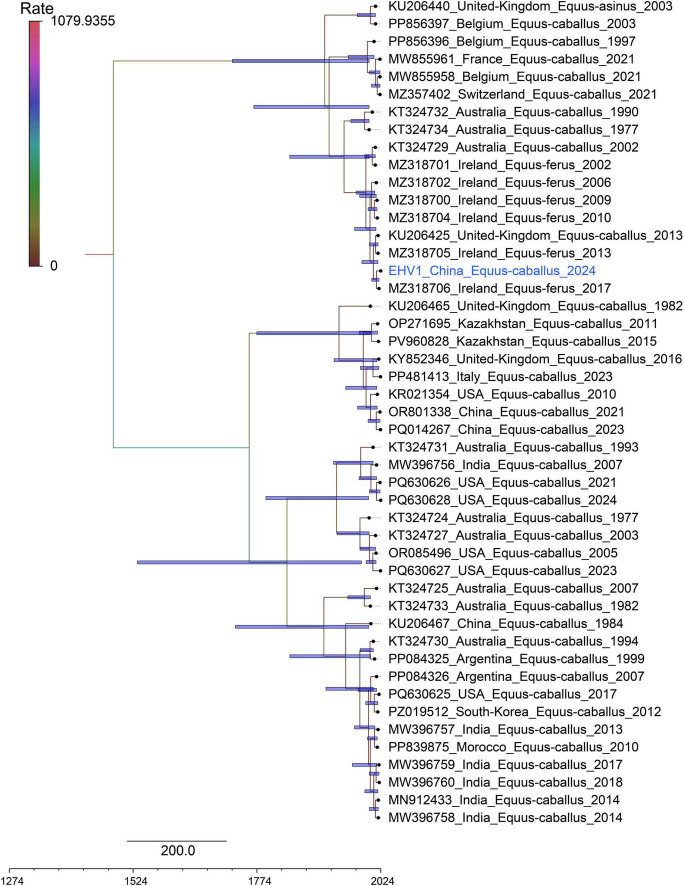
Bayesian time-calibrated phylogenetic tree of EHV-1 based on concatenated ORF30, ORF33, and ORF34 sequences. Posterior probabilities (≥0.90) are indicated at nodes. Scale bar represents evolutionary time in years.

To determine the phylogenetic position of the strain identified in this study (EHV-1/China/2024), representative EHV-1 strains of different years, geographic origins, and hosts (including *Equus caballus*, *Equus ferus*, and *Equus asinus*) were retrieved from GenBank. A Bayesian phylogenetic tree was constructed based on the concatenated ORF30-ORF33-ORF34 sequences (Figure 2).

The Bayesian tree topology revealed that all EHV-1 strains first formed two distinct clades based on host range. Clade A encompassed a broad host spectrum, including *Equus caballus*, *Equus ferus*, and *Equus asinus*, and the EHV-1/China/2024 strain detected in this study was located within this clade. Clade B exhibited strict host specificity, with all strains isolated exclusively from *Equus caballus*.

Within Clade A, EHV-1/China/2024 first clustered with an *Equus ferus*-derived strain isolated in Ireland in 2017 (MZ318706) with a high posterior probability, forming a small monophyletic group. This small clade subsequently clustered with a sister group composed of another Irish *Equus ferus*-derived strain (MZ318705) and a UK *Equus caballus*-derived strain (KU206425). These results indicate that the EHV-1 strain detected in this study belongs to a broadly host-adapted evolutionary clade, shares the most recent common ancestor with an Irish *Equus ferus*-derived strain, and forms a predominantly European-derived evolutionary cluster together with Irish and UK strains from both *Equus ferus* and *Equus caballus*.

## Discussion

In this study, four EHV-1 nucleic acid-positive samples were detected during routine pathogen screening of a batch of imported quarantine horses originating from the Netherlands. Through ORF30 SNP typing, multi-gene amplification and sequencing, and Bayesian phylogenetic analysis, the molecular characteristics and phylogenetic position of this strain were elucidated.

SNP typing and Sanger sequencing confirmed that all four positive samples belonged to the N752 non-neuropathogenic genotype. Consistent with this finding, all positive horses exhibited only mild respiratory signs during quarantine, with no evidence of neurological dysfunction. This observation aligns with the classic conclusion of regarding the association between the D/N752 genotype and neuropathogenicity. However, sporadic cases of EHM caused by N752 strains have been reported in recent years, suggesting inherent limitations in predicting pathogenicity based solely on the ORF30 SNP site (Pusterla et al., 2023). As virus isolation was unsuccessful in this study, the virulence phenotype of this strain could not be directly assessed through *in vitro* or *in vivo* experiments. Therefore, the pathogenicity assessment of this strain still requires comprehensive evaluation combining genotype and clinical presentation, and the potential pathogenic risk posed by N752 strains should not be overlooked.

In terms of phylogenetic analysis, the Bayesian tree constructed based on concatenated multi-sequence alignment revealed a unique evolutionary phenomenon: all reference strains first divided into two distinct clades according to host range. Clade A encompassed three equid species, namely *Equus caballus*, *Equus ferus*, and *Equus asinus*, whereas Clade B was strictly restricted to *Equus caballus* hosts. The strain identified in this study was located within the broadly host-adapted Clade A. This topological structure suggests that during the evolutionary history of EHV-1, certain lineages may have formed closed transmission chains specific to *Equus caballus* under domestication or modern breeding management systems, while Clade A has retained a broader capacity for cross-host transmission. This pattern of divergence has rarely been explicitly reported in previous studies, which may be attributed to the inclusion of more reference sequences from non-*Equus caballus* hosts in this study, thereby enhancing the phylogenetic resolution of host-associated signals. From an evolutionary and epidemiological perspective, broad host tropism implies a more complex maintenance host network for this lineage, and its transmission dynamics at the interface between wild and domestic equids warrant further investigation.

Within Clade A, the strain from this study first formed a monophyletic group with an *Equus ferus*-derived strain isolated in Ireland in 2017 (MZ318706) with high posterior probability, and subsequently clustered with a sister group comprising another Irish *Equus ferus*-derived strain (MZ318705) and a UK *Equus caballus*-derived strain (KU206425), collectively constituting a predominantly European-derived evolutionary cluster. Notably, the horses in this batch were imported from the Netherlands, yet phylogenetic analysis placed this strain within the Ireland/British Isles evolutionary cluster. This geographic discordance between the country of origin and the evolutionary background may be explained in several ways. On the one hand, the Netherlands serves as a major equine trade hub in Europe, with large numbers of horses from Ireland and the United Kingdom being transported through or traded in the Netherlands before export. The strain may have initially entered the Dutch equine population from the Ireland/UK region and subsequently been introduced into China through trade channels. On the other hand, given the relatively limited availability of EHV-1 genomic sequences from the Netherlands in GenBank, the possibility that this evolutionary cluster is also widely distributed in the Netherlands and other parts of northwestern continental Europe cannot be excluded. Therefore, the current phylogenetic inference reflects the evolutionary relationship of the strain rather than simply pointing to its country of import. Furthermore, the strain in this study was more closely related to *Equus ferus*-derived strains than to *Equus caballus*-derived strains, suggesting that its ancestor may have undergone cross-host transmission between *Equus ferus* and *Equus caballus* populations in Europe.

The ORF30, ORF33, and ORF34 gene sequences of the four positive samples in this study were completely identical, indicating a common infection source. Given that the horses belonged to the same batch, this result most likely reflects common exposure within the source herd prior to export, or within-group transmission during transportation and quarantine (Spiesschaert et al., 2015). EHV-1 is primarily transmitted through respiratory droplets and close contact, and stress factors such as transportation and environmental changes can induce reactivation and shedding of latent virus, increasing the risk of within-group transmission. This highlights the importance of EHV-1 prevention and control in the context of international horse transportation and group housing.

In conclusion, this study reports an EHV-1 N752 genotype strain detected in horses imported from the Netherlands, which belongs to a broadly host-adapted evolutionary clade and possesses an Ireland/British Isles evolutionary background. Phylogenetic analysis based on concatenated multi-sequence alignment revealed its close genetic relationship with an Irish *Equus ferus*-derived strain, and simultaneously uncovered a geographic discordance between the evolutionary background of the virus and the direct country of origin of the horses. These findings provide new molecular evidence for understanding the host adaptation divergence and international transmission dynamics of EHV-1, and have reference value for pathogen surveillance in the context of cross-border equine movements.

## Data Availability

The datasets presented in this study can be found in online repositories. The names of the repository/repositories and accession number(s) can be found in the article/supplementary material.
